# The Role of Sodium-Glucose Cotransporter-2 Inhibition in Heart Failure with Preserved Ejection Fraction

**DOI:** 10.3390/pharmacy10060166

**Published:** 2022-11-30

**Authors:** Lindsay Brust-Sisti, Nicole Rudawsky, Jimmy Gonzalez, Luigi Brunetti

**Affiliations:** 1 Department of Pharmacy Practice and Administration, Ernest Mario School of Pharmacy, Rutgers, The State University of New Jersey, 160 Frelinghuysen Road, Piscataway, NJ 08854, USA; 2 Department of Pharmaceutics, Ernest Mario School of Pharmacy, Rutgers, The State University of New Jersey, 160 Frelinghuysen Road, Piscataway, NJ 08854, USA

**Keywords:** heart failure, sodium-glucose transporter 2, dapagliflozin, empagliflozin, ertugliflozin, canagliflozin

## Abstract

Sodium-glucose cotransporter-2 (SGLT2) inhibitors are a novel class of antidiabetic mediations found to also reduce cardiovascular morbidity and mortality and hospitalization for heart failure. Positive results from the EMPEROR-Preserved (empagliflozin) and PRESERVED-HF (dapagliflozin) studies led to recommendations for SGLT2 inhibitors in HFpEF within major international heart failure guidelines. However, studies of ipragliflozin and luseogliflozin, agents approved outside the United States (U.S.), reported different outcomes relative to pivotal trials and failed to realize benefits in the HFpEF population. Varying definitions of HFpEF and outcomes studied complicate the interpretation of study results. SGLT2 inhibitors may cause common adverse events (genital mycotic infections, volume depletion) in addition to rare but severe sequela, including euglycemic diabetic ketoacidosis, Fournier’s gangrene, and lower limb amputation. While evidence of CV benefits grows, SGLT2 inhibitor prescribing has lagged, particularly among patients without diabetes. In the U.S., high cost and administrative hurdles may contribute to decreased patient and clinician uptake of this drug class. Future trial results and clinical experience with SGLT2 inhibitors may lead to expanded use and greater uptake among patients with heart failure.

## 1. Background

Sodium-glucose co-transporter 2 (SGLT2) inhibitors are a class of oral medications originally utilized as antidiabetic drugs. SGLT2 proteins, expressed on the proximal renal tubule, mediate glucose reabsorption from the glomerular filtrate back into the circulation. By inhibiting this transporter, SGLT2 inhibitors increase urinary glucose excretion, thereby reducing blood glucose [1]. The first SGLT2 inhibitor, canagliflozin, was approved by the United States (U.S.) Food and Drug Administration (FDA) in 2013 for adults with type 2 diabetes mellitus (T2DM). Dapagliflozin and empagliflozin were FDA-approved in 2014, followed by ertugliflozin in 2017 [2]. A list of U.S. FDA-approved SGLT2 inhibitors can be found in Table 1.

## 2. Transition into Cardiovascular Space

Following approval for T2DM, researchers sought to investigate the cardiovascular safety benefits of SGLT2 inhibitors, as the effects on cardiovascular morbidity and mortality were unknown. The EMPA-REG OUTCOME investigators evaluated empagliflozin compared to placebo in patients with T2DM and cardiovascular disease. This multicenter trial spanned 42 countries and observed patients for a median time of approximately three years. The primary composite outcome was death from cardiovascular causes, nonfatal myocardial infarction, or nonfatal stroke. Overall, the primary outcome occurred in 10.5% of patients receiving empagliflozin and 12.1% of patients in the placebo group (HR 0.86; 95.02% CI 0.74 to 0.99; *p* = 0.04 for superiority). While this study did not identify significant differences in rates of myocardial infarction or stroke, researchers observed significantly lower rates of death from cardiovascular causes and hospitalization for heart failure. Overall, this study provided evidence that use of empagliflozin was associated with a cardiovascular risk reduction in patients with T2DM [3]. Similarly, the DECLARE-TIMI 58 investigators evaluated dapagliflozin versus placebo in patients with T2DM and atherosclerotic cardiovascular disease. Investigators examined a primary safety outcome of major adverse cardiovascular events (MACE), and dapagliflozin demonstrated noninferiority compared to placebo. The primary efficacy outcomes evaluated MACE and a composite of cardiovascular death or hospitalization for heart failure. While use of dapagliflozin did not reduce MACE, this group did have a significantly lower composite rate of cardiovascular death or hospitalization for heart failure (4.9% vs. 5.8%; HR, 0.83; 95% CI, 0.73 to 0.95; *p* = 0.005). This efficacy finding primarily reflects lower rates of hospitalization for heart failure, as there was not a significant difference seen in cardiovascular death alone [4]. Results from this trial further added to evidence regarding the benefit of SGLT2 inhibitors in cardiovascular disease, namely in the setting of heart failure.

Canagliflozin and ertugliflozin also sought to evaluate cardiovascular outcomes in patients with T2DM and cardiovascular disease or risk factors via the CANVAS trial and VERTIS-CV trial, respectively [5,6]. The CANVAS trial enrolled patients with T2DM and established cardiovascular disease or two or more cardiovascular risk factors and had a median observation time of 3.6 years. The primary outcome, composite of death from cardiovascular causes, nonfatal myocardial infarction, or nonfatal stroke, occurred in 26.9 versus 31.5 participants per 1000 patient years (HR 0.86; 95% CI 0.75 to 0.97; *p* < 0.001 for noninferiority; *p* = 0.02 for superiority). Each individual component of the primary outcome, however, did not reach statistical significance. The results also demonstrated that patients treated with canagliflozin had a lower risk of hospitalization for heart failure, though based on the pre-specified analysis, this finding was not considered to be statistically significant [5]. The VERTIS-CV trial found ertugliflozin was noninferior compared to placebo regarding its primary composite outcome of death from cardiovascular causes, nonfatal myocardial infarction, or nonfatal stroke [6]. The key secondary outcome, composite incidence of cardiovascular death or hospitalization for heart failure, did not differ significantly between the trial groups, a finding that differed compared to the previous studies evaluating SGLT2 inhibitors in cardiovascular outcomes. The individual outcome, hospitalization for heart failure, however, was shown to be lower in the ertugliflozin group (2.5% vs. 3.6%; HR 0.70 [0.54–0.90]), though it was not tested significantly.

With large clinical trials indicating a reduced risk of hospitalization for heart failure with SGLT2 inhibitor therapy, the DAPA-HF Trial Committees, and Investigators sought to explore the effect of dapagliflozin use in patients with New York Heart Association (NYHA) class II–IV heart failure and a reduced ejection fraction (≤40%). Only 45% of the patients enrolled had a diagnosis of T2DM. Over a median time of approximately 18.2 months, the primary outcome, a composite of worsening heart failure or cardiovascular death, occurred in 16.3% of patients in the dapagliflozin group and 21.2% of patients in the placebo group (HR, 0.74; 95% CI 0.65 to 0.85; *p* < 0.001) [7]. This trial demonstrated the benefit of dapagliflozin in heart failure with reduced ejection fraction (HFrEF), regardless of diabetes status, and subsequently led to this agent’s FDA-approval for this broadened indication in May 2020. Dapagliflozin was the first agent in its class to be approved for HFrEF [8].

Empagliflozin was the next medication in this class to receive FDA approval for HFrEF after findings from the EMPEROR-Reduced Trial. This large, randomized controlled trial evaluated empagliflozin in patients with NYHA class II–IV heart failure and an ejection fraction (≤40%). Cardiovascular death or hospitalization for worsening heart failure, occurred in 19.4% of patients in the empagliflozin group compared to 24.7% of patients in the placebo group (HR 0.75; 95% CI, 0.65 to 0.86; *p* < 0.001) [9]. Meta-analysis findings have aligned with these results, demonstrating improved survival and reduced hospitalizations for heart failure with SGLT2 inhibitors [10].

With the mounting evidence supporting SGLT2 inhibitor use in the setting HFrEF, clinical practice guidelines for the management of heart failure were updated to incorporate recommendations for use of these agents. The guidelines include a Class 1 recommendation, “In patients with symptomatic chronic HFrEF, SGLT2 inhibitors are recommended to reduce hospitalization for HF and cardiovascular mortality, irrespective of the presence of type 2 diabetes”. This statement was supported by a Level A quality of evidence [11].

## 3. SGLT2 Inhibitors in Heart Failure with Preserved Ejection Fraction

The elucidated cardiovascular benefits and efficacy in heart failure with reduced ejection fraction catalyzed further investigation regarding the class’s benefit in heart failure with preserved ejection fraction (HFpEF). The definition of HFpEF has been inconsistently categorized as an ejection fraction of >40%, >45%, or ≥50%. Classically, guideline-directed therapy management in HFpEF included optimal control of hypertension, diabetes, and/or cardiovascular disease. Though unclear, there are various molecular and cellular mechanisms by which the SGLT2 inhibitors may work in HFpEF, including:

- Decreased preload (through renal glucose excretion leading to natriuresis and osmotic diuresis) and decreased afterload (through reduced blood pressure and improved vascular function) [12,13,14,15,16];
- Reduced pulmonary artery pressure [13];
- Improved cardiac metabolism and bioenergetics [7,13,14,15,17,18];
- Inhibition of Na^+^/H^+^ exchanger [15,17];
- Reduced left ventricular hypertrophy [14,15];
- Decreased uric acid (marker of oxidative stress) [13,14,15,16];
- Reduced necrosis and cardiac fibrosis [15,17];
- Reduced inflammation [13,15,16,18,19];
- Improved insulin sensitivity [13];
- Weight loss [13,14].

In addition, the positive impact of SGLT2 inhibitors on hypertension, diabetes, and chronic inflammation are beneficial, as all are major risk factors for HFpEF [18].

## 4. Landmark Trials in HFpEF

### 4.1. EMPEROR-Preserved

Large trials have studied the effect of various SGLT2 inhibitors on outcomes in patients with HFpEF. The first to be published, in October 2021, was The Empagliflozin Outcome Trial in Patients with Chronic Heart Failure with Preserved Ejection Fraction (EMPEROR-Preserved) [12]. This study was a randomized, double-blind, placebo-controlled, event-driven, international trial that examined the impact of empagliflozin on major heart failure outcomes in patients with HFpEF, regardless of diabetes mellitus. Patients with LVEF > 40%, New York Heart Association (NYHA) functional class II to IV for ≥3 months prior to enrollment, elevated N-terminal pro–B-type natriuretic peptide (NT-proBNP) adjusted for atrial fibrillation, either structural heart disease within 6 months or heart failure hospitalization within 12 months, and on a steady dose of oral diuretics, if prescribed, were included. They were randomized in a 1:1 ratio to empagliflozin 10 mg once daily (n = 2997) or placebo (n = 2991) plus usual therapy for a median follow up period of 26.2 (IQR, 18.1–33.1) months. The mean patient age was 72 years and 45% were women. Two thirds had a LVEF ≥ 50%, the median LVEF was 54%, and approximately half of the patients had diabetes and an eGFR < 60 mL/min/1.73 m^2^ at baseline.

Empagliflozin significantly reduced the primary outcome, a composite of cardiovascular death or hospitalization for heart failure, which occurred in 415 patients (13.8%) in the empagliflozin group and 511 patients (17.1%) taking placebo (HR, 0.79; 95% CI, 0.69–0.90; *p* < 0.001). The number needed to treat to prevent one primary outcome event was 31 (95% CI, 20–69). The primary composite outcome was primarily led by a lower risk of hospitalization for heart failure which occurred in 259 patients (8.6%) and 352 patients (11.8%) in the empagliflozin and placebo groups, respectively (HR, 0.71; 95% CI, 0.60–0.83). The primary outcome benefit was consistent in those with or without diabetes and the pre-specified ejection fraction subgroups; however, the benefit seemed slightly lessened among patients with LVEF ≥ 60%. There was a nonsignificant 9% lower risk of cardiovascular death with empagliflozin (7.3% vs. 8.2%; HR, 0.91; 95% CI, 0.76–1.09) and no effect on all-cause mortality (14.1% vs. 14.3%; HR, 1.00; 95% CI, 0.87–1.15). Empagliflozin use also caused a significant reduction in total HF hospitalizations (HR 0.73; 95% CI, 0.61–0.88; *p* < 0.001) and decrease in the slope of the eGFR decline (−1.25 ± 0.11 vs. −2.62 ± 0.11; *p* < 0.001) [12]. Pre-specified secondary analysis showed the benefit of empagliflozin on the primary outcome was not significantly different between patients using or not using mineralocorticoid receptor antagonist (MRA) at baseline [20].

To summarize, the EMPEROR-Preserved trial revealed that empagliflozin is superior to placebo in lowering the risk of cardiovascular death and hospitalization in patients with or without diabetes and symptomatic stable HFpEF (EF > 40%). These findings were groundbreaking for the management of HfpEF as this was the first trial to meet its primary endpoint, show clinically meaningful results, and lead to a new treatment option for this population.

### 4.2. DELIVER

Results from the Dapagliflozin Evaluation to Improve the Lives of Patients with Preserved Ejection Fraction Heart Failure (DELIVER) study expanded the role of dapagliflozin to those with HfpEF [19]. DELIVER was a randomized, double-blind, placebo-controlled, event-driven, international trial designed to study the effect of dapagliflozin in reducing the composite of cardiovascular death or heart failure events (hospitalizations or urgent visits). Patients with or without T2DM, with LVEF > 40% and evidence of structural heart disease, and NYHA class II to IV were included. A total of 6263 patients were randomized in a 1:1 ratio to dapagliflozin 10 mg once daily or placebo in addition to concomitant standard of care for up to approximately 39 months [21]. The mean patient age was 72 years and 44% were women. Sixty-six percent of patients had an LVEF ≥ 50%, the mean LVEF was 54.2%; 44.8% had T2DM and 50.1% had eGFR ≥ 60 mL/min/1.73 m^2^ at baseline [19,22].

The DELIVER study results were recently published and showed that dapagliflozin significantly reduced the primary outcome, which occurred in 512 patients (16.4%) in the treatment group and 610 patients (19.5%) in the placebo group (HR, 0.82; 95% CI, 0.73–0.92; *p* < 0.001) over a median of 2.3 years. Worsening heart failure occurred in 368 patients (11.8%) and 455 patients (14.5%) in the dapagliflozin and placebo groups, respectively (HR, 0.79; 95% CI, 0.69–0.91). The primary outcome benefit was consistent among the pre-specified subgroups, including those with or without diabetes and by LVEF (LVEF ≥ 60% and <60%). The risk of death from cardiovascular causes (HR, 0.88; 95% CI, 0.74–1.05) and all causes (HR, 0.94; 95% CI, 0.83–1.07) was not significantly reduced. This clinically meaningful evidence substantiates the use of SGLT2 inhibitors in HFpEF.

## 5. Impact of SGLT2 Inhibitors on Clinical Symptoms in HFpEF

### 5.1. PRESERVED-HF

Findings related to the impact of SGLT2 inhibitors on the burden of HFpEF are also supportive. Dapagliflozin in Preserved Ejection Fraction Heart Failure (PRESERVED-HF) [13], a randomized, double-blind trial of patients with HFpEF (LVEF ≥ 45%), sought to determine if dapagliflozin would improve Kansas City Cardiomyopathy Questionnaire (KCCQ) Clinical Summary Score (KCCQ-CS) compared to placebo. The KCCQ is a patient-reported measure of heart failure symptoms and physical limitations using seven domains (physical limitations, symptom stability, symptom frequency, symptom burden, quality of life, self-efficacy, and social limitations) which are scaled from 0 to 100 points. The symptom frequency and symptom burden domains are joined to determine the total symptom score (TSS); combining symptom frequency, symptom burden, and physical limitation determines the clinical summary score (CS). Lower scores represent more severe symptoms and/or limitations, whereas higher scores indicate better health-related quality of life. A change of five points is considered to be clinically meaningful [23].

In the PRESERVED-HF study, patients were randomized to dapagliflozin (n = 162) or placebo (n = 162) for 12 weeks. The median patient age was 70 years and 57% were women. Median LVEF was 60% and 56% had T2DM. The baseline KCCQ-CS was 63.4 ± 19.7 in the dapagliflozin group and 61.8 ± 20.3 in the placebo group. The study was powered to detect a 4.7-point change in mean KCCQ-CS between groups at 12 weeks. Dapagliflozin improved symptoms and physical limitations, measured by KCCQ-CS at 12 weeks (effect size, 5.8 points; 95% CI, 2.3–9.2; *p* = 0.001). Findings were consistent within pre-specified subgroups, including baseline LVEF ≤ 60% or >60% [13]. By showing significantly improved symptoms and physical limitations, the results from the PRESERVED-HF trial help to support the use of SGLT2 inhibitors for the management of HFpEF.

### 5.2. CHIEF-HF

Though canagliflozin is not currently FDA-approved for heart failure, researchers sought to investigate this agent’s impact on symptoms of HFpEF. The Kansas City Cardiomyopathy Questionnaire (KCCQ) Total Symptom Score (TSS) was also explored in A Study on Impact of Canagliflozin on Health Status, Quality of Life, and Functional Status in Heart Failure (CHIEF-HF) [24], a randomized, double-blind, placebo-controlled, decentralized, virtual study conducted in the U.S. Stratified by ejection fraction (HFrEF, EF < 40%; HFpEF, EF > 40%), patients were randomized in a 1:1 ratio to canagliflozin 100 mg daily (n = 222) or matching placebo (n = 226) for 12 weeks. The mean patient age was 63.4 years and 45% were women. Sixty percent of patients had HFpEF and 28% had T2DM. The baseline KCCQ TSS was 57.4 ± 21.3 in the canagliflozin group and 58 ± 21.1 in the placebo group. The study was powered to detect a mean change from baseline of 3 points in the KCCQ TSS between the two groups at 12 weeks and enrollment was stopped early by the sponsor. At 12 weeks, KCCQ TSS scores improved to 67.1 ± 22 and 63.2 ± 22 in the canagliflozin and placebo groups, respectively. The mean difference in change at 12 weeks was 4.3 points (95% CI, 0.8–7.8; *p* = 0.016) and canagliflozin was determined to be superior to placebo in helping patient-reported heart failure symptoms. Similar findings were observed in patients regardless of type of heart failure (HFpEF LS mean difference 4.5; 95% CI, −0.3–9.4) or diabetes status [24]. The findings from this study impact the care of patients with HFpEF and further add to literature regarding the SGLT2 inhibitors’ impact on symptoms and quality of life.

## 6. Globally Approved SGLT2 Inhibitors

Findings from large trials regarding the benefits of SGLT2 inhibitors in HFpEF are also supported by globally used products. Sotagliflozin is a dual sodium-glucose co-transporter-2 and 1 (SGLT2/1) inhibitor that is no longer authorized for marketing in the European Union [25], The Effect of Sotagliflozin on Cardiovascular Events in Patients with Type 2 Diabetes Post Worsening Heart Failure (SOLOIST-WHF Trial) was a randomized, double-blind, placebo-controlled, event driven trial evaluating the safety and efficacy of SGLT2 inhibitors in patients with type 2 diabetes and recent hospitalization for worsening heart failure [14]. Patients were randomized to sotagliflozin 200 mg once daily (with possible up titration to 400 mg) (n = 608) or placebo (n = 614) for a median follow up of 9 months. The median patient age was 70 years and 33.7% were women. Seventy nine percent of patients had a LVEF < 50%; the median LVEF was 35%.

Sotagliflozin initiated before or shortly after discharge resulted in a significantly lower total number of cardiovascular deaths and hospitalizations and urgent visits for heart failure (245 events) than placebo (355 events) (51 vs. 76.3 events per 100 patient years; HR 0.67, 95% CI, 0.52–0.85; *p* < 0.001). The primary outcome finding was primarily driven by reduced hospitalizations and urgent visits (40.4% vs. 63.9%; HR 0.64; 95% CI, 0.49 to 0.83). The finding was consistent among the subgroup of patients with preserved left ventricular ejection fraction (LVEF ≥ 50%) with 30.6 vs. 64 events per 100 patient years in the sotagliflozin and placebo groups, respectively (HR 0.48; 95% CI, 0.27–0.86); however, early trial termination due to loss of funding and the small subgroup sample (n = 256) limit sound conclusions for this population. The trial, however, did not show a reduction in rate of cardiovascular death or death from any cause [14]. In brief, SOLOIST-WHF is significant and impacts clinical practice as it was the first trial to show that SGLT2/1 inhibitors are effective and safe for use in those with T2DM, reduced or preserved ejection fraction heart failure, and recent heart failure admission.

Ipragliflozin and luseogliflozin are other SGLT2 inhibitors approved outside the U.S. for the treatment of T2DM. These agents have been evaluated in the setting of HFpEF; however, studied other primary outcomes as compared to the previously mentioned landmark trials. The EXCEED study was an open-label, multicenter, randomized, two-arm interventional trial that compared changes in diastolic function in 68 Japanese patients with T2DM and HFpEF (≥50%). Results showed no improvement in left ventricular diastolic function at 24-weeks with ipragliflozin versus conventional treatment, though study limitations impact generalizability [26]. Luseogliflozin’s effect on HFpEF (LVEF > 45%) in 173 patients with diabetes mellitus was evaluated in the open-label, multicenter, randomized MUSCAT-HF trial. Results showed no significant difference in the amount of reduction in BNP concentrations, a surrogate biomarker, after 12 weeks between luseogliflozin and voglibose [27]. Study design limitations and lack of statistical power impact these findings [27]. These trials did not identify a benefit of these SGLT2 inhibitors in HFpEF based on their respective primary outcomes; however, their impact on hospitalizations for heart failure are not known and further studies are needed.

## 7. Study Comparison

The EMPEROR-Preserved and DELIVER trials are large landmark trials with similar designs that will establish the role of empagliflozin and dapagliflozin in patients with HFpEF. The DELIVER trial is unique in its inclusion of patients with heart failure with recovered ejection fraction (patients with any prior LVEF ≤ 40%) and those with acute decompensated heart failure requiring intravenous heart failure therapies or mechanical support [22]. It is important to know that the definition of HFpEF varies in trials with study eligibility at LVEF > 40%, ≥45%, and >50% for the EMPEROR-Preserved [12] and DELIVER [22], PRESERVED-HF [13], and SOLOIST-WHF [14] trials, respectively. Select baseline characteristics are detailed in Table 2 and the use of concomitant evidence-based heart failure therapies is compared in Table 3. Across the trials, patients were similar in age and predominantly of White race. The majority of patients were also taking an angiotensin-converting enzyme inhibitor (ACE) or angiotensin receptor blocker (ARB); mineralocorticoid receptor antagonist (MRA) use was greatest in the SOLOIST-WHF trial. Primary efficacy outcomes for the trials are summarized in Table 4 and Table 5.

## 8. Real World Evidence

Using a Swedish heart failure registry, Thorvaldsen et al. showed that real-world eligibility for empagliflozin and dapagliflozin in the heart failure population ranges from 30–50% when applying clinical trial selection criteria, is 75% using a pragmatic, “clinically relevant” approach, and 74–81% when applying regulatory labelling. The authors hope this data can contribute to future heart failure trial design and inclusion criteria [28]. Additionally, the EMPULSE trial was designed to address providers’ reluctance to prescribe SGLT2 inhibitors in the acute setting, despite supportive results for chronic heart failure. It was a double-blind, randomized, trial in 530 patients hospitalized with acute heart failure. The primary endpoint was a composite of death from any cause, number of heart failure events, time to first heart failure event, and the change in baseline KCCQ TSS. The study showed that empagliflozin 10 mg once daily provided statistically significant and clinically meaningful benefit in patients hospitalized with heart failure, regardless of ejection fraction or diabetes status, within 90 days after randomization. Specifically, empagliflozin treated patients were 36% more likely to see the clinical benefit compared to placebo (stratified win ratio, 1.36; 95% CI, 1.09–1.68; *p* = 0.0054), supporting initiation of empagliflozin in addition to standard care in patients hospitalized for acute heart failure [29].

## 9. SGLT2 Inhibitor Place in Therapy: HFpEF

The 2022 AHA/ACC/HFSA Guideline for the Management of Heart Failure recommends that SGLT2 inhibitors “can be beneficial in decreasing HF hospitalizations and cardiovascular mortality” in patients with HFpEF (LVEF ≥ 50%) (Class of Recommendation 2a) [11]. Trials with SGLT2 inhibitors in HFpEF were ongoing at the time of the latest European Society of Cardiology (ESC) Guideline publication (2021); therefore, no specific recommendations for this population were made [30]. Empagliflozin is FDA-approved in the U.S. and EMA-authorized in Europe for HFpEF at a dose of 10 mg once daily [31,32]. At the time of writing, dapagliflozin has an “off-label” use in the U.S. for HFpEF in combination with other evidence-based therapies at a dose of 10 mg once daily [13].

## 10. Safety Considerations

Common adverse events (AE) reported with SGLT2 inhibitor use include increased risk of infections (genital and mycotic) and volume depletion [33]; some of these events are driven by physiological glycosuria induced during SGLT2 inhibitor therapy and may be the impetus for therapy discontinuation in some. Interestingly, common AEs were not described in the EMPEROR-Preserved trial, though Nassif and colleagues’ open label study saw about 11.1% of patients randomized to dapagliflozin for HFpEF has AEs contributing to discontinuation [13]. Moreover, an analysis of the UK CPRD primary care database found an increased risk genital infection with SGLT2 inhibitors compared to DPP4 inhibitors (HR 3.64, 95% CI 3.23–4.11), though a similar magnitude of discontinuation risk within the first year compared to DPP4 inhibitors [34]. More rare and serious adverse events have been documented in pivotal randomized controlled trials and further explored in reports throughout the literature ranging from case studies to registry analyses.

## 11. Euglycemic Diabetic Ketoacidosis

The U.S. FDA publicly recognized the potential of euglycemic diabetic ketoacidosis (euDKA) with SGLT2 inhibitors in a Drug Safety Communication published in 2015 based on postmarketing surveillance data obtained through the FDA Adverse Event Reporting System (FAERS) database [35]. Defined as diabetic ketoacidosis with plasma glucose levels < 300 mg/dL, euDKA occurs in patients on SGLT2 inhibitors due to a potent augmentation of the urinary glucose clearance rate. Whereas both conditions stimulate ketosis through a lower insulin-to-glucagon ratio, SGLT2-induced euDKA is characterized by a nearly doubled renal glucose clearance rate [36]. The estimated risk of acidosis varies depending on study design but has been reported to range from 3.7 times greater than other antihyperglycemics based on meta-analysis of published literature on euDKA related to SGLT2 inhibitors to 14 times greater than dipeptidyl peptidase 4 (DPP4) inhibitors from analysis of the FAERS database [37,38]. Clinically, patients should be closely monitored in circumstances of heightened risk of ketoacidosis (e.g., reduce oral intake, insulin reductions, illness), which may also necessitate temporarily discontinuing SLGT2 inhibitors (e.g., perioperatively).

## 12. Fournier’s Gangrene

The U.S. FDA also announced a warning in 2018 of cases of Fournier’s gangrene among patients taking SGLT2 inhibitors, necessitating inclusion of this adverse effect into their medication guides [39] Since the initial case reports submitted to the FDA, multiple randomized controlled trials have tracked the incidence of Fournier’s gangrene. One meta-analysis of 84 trials, enrolling over 40,000 type 2 diabetic patients found no difference in the incidence of this adverse effect compared to placebo or other antidiabetic comparators (OR 0.90, 95% CI 0.71–1.13) [40]. An analysis of the IBM MarketScan commercial insurance database in the U.S. also failed to identify a significantly higher adjusted risk of Fournier’s gangrene compared to DPP4 inhibitor use (aHR 0.25, 95% CI 0.04–1.74); however, a higher point estimate of risk was noted compared to all non-SGLT2 inhibitor antihyperglycemic medications across diagnosis settings (aHR 1.80, 95% CI 0.53–6.11), leading the authors to conclude uncertainty exists in the risk of this infection against the backdrop of non-SGLT2 inhibitor antidiabetic medications [41].

## 13. Lower Limb Amputation

The pivotal CANVAS study of canagliflozin was the first study to mark a higher risk of lower limb amputation (HR 1.97, 95% CI: 1.41–2.75), which led to the issuance of a black box warning (BBW) on its prescribing information for this adverse effect [5]. As of 2020, no SGLT2 inhibitor carries a BBW for lower limb amputation as the BBW was removed following additional data availability. Meta-analyses of available RCTs and observational studies of SGLT2 inhibitors indicate no significant association between exposure and amputation (RR 1.28, 95% CI: 0.93–1.76) using a random effects model; however, significant heterogeneity was present (I^2^ = 62.0%) and subgroup analysis of canagliflozin vs. placebo re-demonstrated an increased risk (RR 1.59, 1.26–2.01) [42]. Real-world data from multiple U.S. administrative claims databases further support the claim that SGLT2 inhibitors do not increase risk of lower amputations (HR 0.75, 95% CI: 0.40–1.41) [43]. Similarly, analysis of over 3 million medical records found no increase in amputations between SGLT2 inhibitors and incretins, instead seeing lower limb amputation risk driven by pre-existing peripheral artery disease [44].

## 14. Patient Access Considerations

### Prescribing Trends

Multiple studies ranging in scope from local health systems to large claims databases have demonstrated low but growing community uptake of SGLT2 inhibitors across various populations despite significant morbidity and mortality reduction associated with their use. Using the IQVIA National Prescription Audit, Adhikari et al. reported a total of 63.2 million prescriptions dispensed for SGLT2 inhibitors between January 2015 and December 2020 [45], corresponding to an annual growth in prescriptions of 15.6% during this time frame. From a different perspective, one analysis of a national cohort of U.S. clinicians servicing Medicare beneficiaries saw SGLT2 inhibitor prescribing prevalence increase from 4.3% in 2014 to 19.5% in 2018 among metformin-prescribing providers, thus indicating over 80% of providers did not write orders for this drug class. Of note, the percentage of clinicians prescribing SGLT2 inhibitors increased between 2014 to 2018 across prescriber specialty, with the largest gains among endocrinologists and cardiologists [46].

Low utilization of SGLT2 inhibitor therapy has also been described within the heart failure population, despite recent FDA approval for this indication and first-line recommendations from multinational heart failure guidelines. A review of prescribing patterns among cardiologists at the University of Mississippi Medical Center between 2013 and 2019 found SGLT2 inhibitors prescribed in just 1.4% of patients with T2DM and cardiovascular disease [47]. A drug utilization review of Optum Clinformatics insurance data found the overall proportion of patients prescribed SGLT2 inhibitors with cardiovascular disease or HF only increased by 3.4% from 2013 to 2018 [48]. Internationally, a cross-sectional analysis of diabetic patients within a British general practice research database found only 11.0% of the population (N = 242,624) were prescribed an SGLT2 inhibitor of which just 4.3% possessed a comorbid heart failure diagnosis [49]. Similarly, low SGLT2 inhibitor prescribing volume has been documented in the CKD population. Cross-sectional analysis of the Mass General Brigham (MGB) CKD registry in 2021 found only 6% of patients with comorbid diabetes and 0.3% of those without diabetes received a prescription [50].

## 15. Insurance Coverage and Affordability

The low use of SGLT2 inhibitors may be due to multiple reasons stemming from both prescriber perspectives such as unfamiliarity or clinical inertia to patient concerns over health insurance coverage and affordability. Medication access varies with the structure and administration of health care systems found across the globe, but formulary inclusion and out-of-pocket (OOP) costs often impact availability and affordability for patients. Formulary coverage varies in the U.S. depending on the payor; from the U.S. perspective, private insurance and Medicare insurance enrollment is associated with improved medication adherence relative to Medicaid or uninsured status [51].

Plan level analysis of commercial, employer-based, and U.S. Medicaid insurance plans found broad coverage for at least one SGLT2 inhibitor and consistent cost-sharing strategies for beneficiaries—co-payment (approximately $40) or co-insurance (between 20–40%). Prerequisites like prior authorizations or step therapy requirements were commonly employed by Medicaid plans and commercial or employer-sponsored plans, respectively, which may serve as barriers to prescribing [52]. A cross-sectional review of U.S. Medicare Part D plans in the first quarter of 2019 saw widely disparate coverage of individual SGLT2 inhibitors (empagliflozin at 95.4% to ertugliflozin at 5.5%, without prior authorization); despite high coverage, the estimated median annual OOP costs for empagliflozin were $1097 (IQR, $932–1271) [53]. In a single center analysis, researchers noted nearly 80% of cases required provider interventions for insurance authorization, appeals, financial assistance, or side effect management, and the median monthly cost for SGLT2 inhibitor and/or GLP-1 RA therapy was $70.50 following insurance approvals [54]. Taken together, patients may still face significant financial burdens from OOP costs despite seemingly adequate insurance coverage of SGLT2 inhibitors.

While cost and insurance access may vary depending on individual plan specifics and patient characteristics, we know higher incurred costs and lack of coverage negatively impact medication adherence; any benefits attributed to medication therapy will not fully materialize in circumstances of medication nonadherence. For instance, medication nonadherence was reported by 52% of surveyed uninsured patients in one community cross-sectional study in the state of Georgia [55]. Patients reporting insufficient financial means are also more significantly likely to not take medication as prescribed (e.g., skip doses, delay refills) [51]. Higher OOP costs have been tied to decreased adherence—one study demonstrated decreased antidiabetic medication adherence after monthly costs exceeded $33 [56].

To mitigate affordability concerns, manufacturers of SGLT2 inhibitors offer patient assistance programs with varying eligibility criteria. Specific details with each program vary depending on manufacturer, but most offer coupons or vouchers to assist patients with commercial (non-government provided) insurance plans or to those meeting a financial threshold who are uninsured. No studies have been published to date specifically reviewing outcomes with programs related to SGLT2 inhibitor medication access; however, literature exists on the impact these programs provide in general. Enrollment in these manufacturer-sponsored financial medication assistance programs significantly reduced abandonment of an initial prescription fill (RR 0.12, 95% CI 0.08–0.18) and discontinuation of therapy thereafter (HR 0.76, 95% CI 0.66–0.88) [57]. Similarly, pharmacy or clinic-based medication assistance programs have also demonstrated the ability to improve outcomes such as glycated hemoglobin and adherence in a meta-analysis of studies within a diabetic population [58]. Some manufacturers also participate in the federal 340B Drug Pricing Program, allowing safety-net hospitals (e.g., those with a disproportionate share of patients without insurance or with Medicaid, and to rural hospitals) to purchase outpatient medications at much lower prices. Overall, strategies to defray medication costs or expand coverage would be anticipated to promote access and appropriate use of medications, including SGLT2 inhibitors.

## 16. Conclusions

SGLT2 inhibitors have altered the landscape of treatment options in patients with heart failure with preserved ejection fraction. Sufficient data exist to support the incorporation of these ground-breaking therapies to enhance patient outcomes. Strategies to promote patient accessibility to these therapies should be considered a priority.

## Figures and Tables

**Table 1:** Summary of FDA-approved SGLT2 inhibitors.

Generic Name	Brand Name	Dose (mg)	Frequency	FDA-Approved Indication(s)
Canagliflozin	Invokana^®^	100, 300	Once daily	Type 2 diabetes mellitus
Dapagliflozin	Farxiga^®^	5, 10		Type 2 diabetes mellitus, Chronic kidney disease, Heart failure with reduced ejection fraction
Empagliflozin	Jardiance^®^	10, 25		Type 2 diabetes mellitus, Heart failure with preserved or reduced ejection fraction
Ertugliflozin	Steglatro^®^	5, 15		Type 2 diabetes mellitus

**Table 2:** Baseline Characteristics in Landmark Clinical Trials Evaluating SGLT2 inhibitors in Heart Failure with Preserved Ejection Fraction.

Characteristic	EMPEROR-Preserved [12]	PRESERVED-HF [13]	SOLOIST -WHF [14]	DELIVER [21,22]	CHIEF-HF [24]
	Empagliflozin (n = 2997)	Dapagliflozin (n = 162)	Sotagliflozin (n = 608)	Dapagliflozin (n = 3131)	Canagliflozin (n = 222)
Age—yr	71.8 ± 9.3 ^a^	69 (64–77) ^b^	69 (63–76) ^b^	71.8 ± 9.6 ^a^	62.9 ± 13.19 ^a^
Female sex ^c^	1338 (44.6)	92 (56.8)	198 (32.6)	1364 (43.6)	104 (46.8)
Male sex ^c^					
White race ^c^	2286 (76.3)	108 (67.1)	567 (93.3)	2214 (70.7)	182 (82.0)
Black or African American race ^c^	133 (4.4)	50 (31.1)	25 (4.1)	81 (2.6)	35 (15.8)
North America Location ^c^	360 (12.0)		39 (6.4)	428 (13.7)	
NYHA I ^c^	3 (0.1)				
NYHA II ^c^	2432 (81.1)	96 (59.3)		2314 (73.9)	
NYHA III ^c^	552 (18.4)	65 (40.1)		807 (25.8)	
NYHA IV ^c^	10 (0.3)			10 (0.3)	
LVEF—%	54.3 ± 8.8 ^a^	60 (55–65) ^b^	35 (28–47) ^b^	54.0 ± 8.6 ^a^	
LVEF > 40% to <50% ^c^	995 (33.2)				
LVEF < 50% ^c^			481 (79.1)	1067 (34.1)	
LVEF ≥ 50% to <60% ^c^	1028 (34.3)			1133 (36.2)	
LVEF ≥ 60% ^c^	974 (32.5)			931 (29.7)	
NT-proBNP—pg/mL ^b^	994 (501–1740)	641 (373–1210)	1816.8 (854.7–3658.5)		
Hospitalization for heart failure during previous 12 months ^c^	699 (23.3)				
Previous hospitalization for heart failure ^c^		98 (60.5)			
Diabetes mellitus history ^c^	1466 (48.9)	90 (55.6)		1401 (44.7)	66 (29.7)
eGFR—mL/min/1.73 m^2^	60.6 ± 19.8 ^a^	56 (42–69) ^b^	49.2 (39.5–61.2) ^b^	61 ± 19 ^a^	
eGFR < 60 mL/min/1.73 m^2,d^	1504/2997 (50.2)				
eGFR ≥ 60 mL/min/1.73 m^2,c^				3138 (50.1)	

eGFR = estimated glomerular filtration rate; NT-proBNP = N-terminal pro B-type natriuretic peptide NYHA = New York Heart Association; LVEF = left ventricular ejection fraction. ^a^ mean ± SD. ^b^ median (IQR). ^c^ number (percent). ^d^ number/total number (percent).

**Table 3:** Concomitant Medications in Landmark Trials Assessing the Efficacy of SGLTi in Heart Failure with Preserved Ejection Fraction.

Drug Class	PRESERVED-HF [13]	SOLOIST-WHF [14]	DELIVER [21,22]	EMPEROR-Preserved [28]
ACEi, %	61 ^a^	41	33	40
ARB, %		42	34	39
ARNI, %	2	17	4	2
MRA, %	36	65	39	37

ACE = angiotensin-converting enzyme inhibitor; ARB = angiotensin receptor blocker; ARNI = angiotensin receptor-neprilysin inhibitor; MRA = mineralocorticoid receptor antagonist. ^a^ Reported as ACEi/ARB.

**Table 4:** Summary of Efficacy Outcomes in Landmark Trials Evaluating SGLT2 inhibitors in Heart Failure with Preserved Ejection Fraction.

	Composite of CV Death or HF Hospitalization	Composite of Worsening HF (Hospitalization or Urgent Visit) or CV Death	Composite of CV Death and HF Hospitalizations and Urgent Visits	First HF Hospitalization	CV Death	Any Death
EMPEROR-Preserved [12] (N = 5988)	HR 0.79; 95% CI, 0.69–0.90; *p* < 0.001			HR 0.71; 95% CI, 0.60–0.83	HR 0.91; 95% CI, 0.76–1.09	HR 1.00; 95% CI, 0.87–1.15
SOLOIST-WHF [14] (N = 1222)			HR 0.67; 95% CI, 0.52–0.85; *p* < 0.001		HR 0.84; 95% CI, 0.58–1.22	HR 0.82; 95% CI, 0.59–1.14
DELIVER [21] (N = 6263)		HR 0.82; 95% CI, 0.73–0.92; *p* < 0.001			HR 0.88; 95% CI, 0.74–1.05	HR 0.94; 95% CI, 0.83–1.07

CV = cardiovascular; HF = heart failure.

**Table 5:** Summary of Clinical Symptom Outcomes in Landmark Trials Evaluating SGLT2 inhibitors in Heart Failure with Preserved Ejection Fraction.

	KCCQ-CS at 12 Weeks	KCCQ TSS at 12 Weeks
PRESERVED-HF [13] (N = 324)	Effect size, 5.8 points; 95% CI, 2.3–9.2; *p* = 0.001	
CHIEF-HF [24] (N = 448)		Mean difference, 4.3 points; 95% CI, 0.8–7.8; *p* = 0.016

KCCQ-CS = Kansas City Cardiomyopathy Questionnaire Clinical Summary Score; KCCQ TSS = Kansas City Cardiomyopathy Questionnaire Total Symptom Score.

## Data Availability

No datasets were used to prepare this publication.
